# A Profile of Adult Severe Acute Respiratory Syndrome Coronavirus 2 Pneumonia Patients According to Pneumococcal Vaccination Status

**DOI:** 10.3390/vaccines11111630

**Published:** 2023-10-24

**Authors:** María Morales-Suárez-Varela, Diana Toledo, María Amelia Fernández-Sierra, María Liébana, Gerardo Rubiera, Gema Navarro, Concepción Prados, Judith Chamarro, Isabel Peraita-Costa, Angela Domínguez, Working Group of Project FIS PI19/00354

**Affiliations:** 1 Research Group in Social and Nutritional Epidemiology, Pharmacoepidemiology and Public Health, Department of Preventive Medicine and Public Health, Food Sciences, Toxicology and Forensic Medicine, Faculty of Pharmacy, Universitat de València, Av. Vicent Andrés Estelles s/n, Burjassot, 46100 València, Spain; isabel.peraita@uv.es; 2 Biomedical Research Center in Epidemiology and Public Health Network (CIBERESP), Carlos III Health Institute, Av. Monforte de Lemos 3-5 Pabellón 11 Planta 0, 28029 Madrid, Spain; dtoledo@ub.edu (D.T.); angela.dominguez@ub.edu (A.D.); 3 Department of Medicine, Faculty of Medicine and Health Sciences, Universitat de Barcelona, C/Casanova 143, 08036 Barcelona, Spain; maria.liebana.sspa@juntadeandalucia.es; 4 Hospital Universitario Virgen de las Nieves, Av. de las Fuerzas Armadas 2, 18014 Granada, Spain; mamelia.fernandez.sspa@juntadeandalucia.es; 5 Hospital Universitario de Cabueñes, C/de Los Prados 395, 33394 Gijón, Spain; gerardo.rubiera@sespa.es; 6 Parc Tauli Hospital Universitari, C/Parc Taulí 1, 08208 Sabadell, Spain; gnavarro@tauli.cat; 7 Institut d’Investigació i Innovació Parc Tauli (I3PT-CERCA), Plaça Torre de l’Aigua s/n, 08208 Sabadell, Spain; 8 Department of Paediatrics, Obstetrics and Gynaecology and Preventive Medicine and Public Health, Faculty of Medicine, Universitat Autònoma de Barcelona, Av. Can Domènech Edifici M, 08193 Bellaterra, Spain; 9 Hospital Universitario La Paz, P.º de la Castellana 261, 28046 Madrid, Spain; conchaprados@gmail.com; 10 Hospital Universitario de Navarra, C/Irunlarrea 3, 31008 Pamplona, Spain; judith.chamorro.camazon@cfnavarra.es

**Keywords:** SARS-CoV-2 pneumonia, *Streptococcus pneumoniae*, pneumococcal vaccine

## Abstract

Certain patient profile characteristics, such as preexisting medical conditions, can modify the risk of developing SARS-CoV-2 pneumonia among adults vaccinated and not vaccinated against pneumococcal disease. This retrospective cohort study aimed to quantify the risk of pneumonia caused by SARS-CoV-2 among individuals from 15 to 64 years old with and without pneumococcal vaccination in Spain during the 2020–2021 influenza season and establish a risk profile of patients more likely to develop SARS-CoV-2 pneumonia. Data (demographic information, patient medical history, and lifestyle habits) were gathered both directly from the patient via personal interview and by reviewing electronic medical records. In an adjusted analysis for pneumococcal vaccinated patients, visits to hospital outpatient clinics were protective while visits to primary health care services, being widowed, obese, and not using masks in outdoor open spaces were identified as risk factors. For patients who had not received a pneumococcal vaccine, visits to hospital outpatient clinics were protective, while being overweight or obese, alcohol consumption, and not using masks in outdoor open spaces were identified as risk factors. Concerning comorbidities, in the pneumococcal vaccinated group none were found to be protective but having diabetes or other respiratory diseases were identified as risk factors. In the unvaccinated group, undergoing immunosuppressive treatment and having metastatic tumors were protective factors, while cerebrovascular disease and obesity with a BMI ≥ 40 were risk factors. A similar risk profile for developing SARS-CoV-2 pneumonia in pneumococcal vaccinated and non-vaccinated individuals was found. Generally, vaccinated individuals had a lower risk of developing SARS-CoV-2. The findings suggest that vaccination against *S. pneumoniae* could prevent and reduce SARS-CoV-2 pneumonia. Additionally, this study has identified individuals with other medical conditions, such as obesity, underweight, diabetes, and a history of respiratory diseases, who are at an increased risk of developing SARS-CoV-2 pneumonia and could benefit from vaccination and supervision.

## 1. Introduction

Although in most cases of community-acquired pneumonia (CAP) a responsible pathogen is not usually identified, the main etiologic agents are *Streptococcus pneumoniae* and viruses [1]. Distinguishing viral pneumonia from bacterial pneumonia is difficult in the community. However, there may be important clues in the history and the examination that can help to differentiate them [2].

*Streptococcus pneumoniae* infection is a significant cause of morbidity and mortality. Pneumococcal pneumonia is the most common disease caused by *S. pneumoniae*. It can present as either invasive pneumococcal disease (IPD), in combination with bacteremia (bloodstream infection) and/or meningitis (infection of the meninges that surround the brain), or as non-invasive pneumococcal pneumonia (PP) if it occurs alone [3,4]. Both are caused by infection with the same bacteria but produce different signs and symptoms. Symptoms include abrupt onset of fever, shaking chills or rigors, chest pain, cough, shortness of breath, rapid breathing and heart rate, and weakness. The case fatality rate is 5–7% and may be much higher in older adults. 

Viruses are becoming increasingly important as etiologic agents of pneumonia, mainly due to improved diagnostic techniques, and now account for approximately one-third of community-acquired pneumonia cases [1]. Viruses that infect the upper respiratory tract may also cause pneumonia. SARS-CoV-2, the virus that causes the current COVID-19 pandemic with devastating consequences, and the influenza virus are the most common causes of viral pneumonia in adults [5].

SARS-CoV-2 pneumonia can be severe, causing low levels of oxygen in the blood and leading to respiratory failure, and in many cases a condition called acute respiratory distress syndrome. Viral pneumonia caused by the SARS-CoV-2 virus generally occurs in both lungs. As the lungs fill with fluid, oxygen exchange becomes more difficult and results in breathing difficulties [5]. Research suggests that SARS-CoV-2 pneumonia spreads across the lungs slowly, using the immune system to spread, which means it tends to last longer and causes damage in more places [6]. Recovery may take months before symptoms cease [5,7]. 

Pneumococcal vaccination is an important preventive health care measure that substantially reduces the burden of pneumococcal disease in vaccinated individuals and the population. Pneumococcal vaccination is indicated for adults < 65 with risk factors for pneumococcal disease or severe adverse outcomes of the disease. 

Two types of pneumococcal vaccines are available for clinical use: the pneumococcal polysaccharide vaccine (PPSV) and the pneumococcal conjugate vaccine (PCV). The PPSV is composed of partially purified pneumococcal capsular polysaccharides. The only available formulation contains 23 pneumococcal polysaccharides (PPSV23) from the 23 serotypes that were the most common cause of pneumococcal disease in adults in the 1980s. 

There is a debate regarding the appropriate use of the pneumococcal vaccine in adults, and no universal consensus on vaccination recommendations exists [8,9,10,11]. Recommendations vary depending on the entities that emit them and have also changed over time. 

In January 2022, the CDC updated its recommendations and now recommends pneumococcal vaccination for adults 19–64 years old who have certain chronic medical conditions or other risk factors [12]. In Spain, these recommendations have been adopted by the different autonomous communities, and currently one polysaccharide vaccine (PPSV23) and four conjugate vaccines (PCV10, PCV13, PCV 15, and PCV20) are available [13,14,15,16].

Although the upward trend observed in the incidence of IPD before the COVID-19 period may have been influenced by changes in the reporting system [17,18,19], its decrease during the COVID-19 period [20] may have been influenced by factors other than vaccination such as social distancing, hand washing, and mask wearing [21,22,23]. Given the available data, it would not be possible to rule out the potential waning impact of vaccination programs. Clear evidence regarding the effectiveness of vaccination for this population group remains elusive [16,24,25].

Given these circumstances and the lack of data on the profile of vaccinated patients who still develop pneumonia, there is a need to study the factors related to the profile of patients aged 15–64 in Spain who would be more and less protected by vaccination against pneumonia.

The main aim of this study was to identify the risk profile of patients aged 15–64 years who develop SARS-CoV-2 pneumonia and ascertain if it is affected by pneumococcal vaccination status. 

## 2. Materials and Methods

### 2.1. Design and Study Population

A multi-center retrospective cohort of patients 15 and 64 years old with SARS-CoV-2 pneumonia. Participants were enrolled from the 40th week of 2020 until the 20th week of 2021.

The study protocol received approval from the University of Barcelona Institutional Review Board for research involving human subjects (IRB00003099). The Ethic Committee of the hospitals involved approved the study. In accordance with the Declaration of Helsinki, all enrolled patients provided either verbal or written informed consent during their assessment, confirming that their data could be used for research purposes. For participants under the age of 18, informed consent was provided by a parent and/or legal guardian.

After obtaining informed consent, demographic information such as age, height, weight, gender, and employment data was collected. Patient medical history was also compiled, including but not limited to a history of diabetes, cardiopulmonary conditions, heart attack, stroke, hypertension, hypothyroidism, hyperlipidemia, rheumatoid arthritis, asthma, cancer, any autoimmune conditions, and body mass index (BMI) categorized as underweight (BMI < 18.5), normal weight (BMI 18.5–24.9), overweight (BMI 25.0–29.9) and obese (BMI > 30). The Charlson comorbidity index [26] was calculated for each patient. Lifestyle habits including hygiene practices, smoking, mask usage, and alcohol consumption were evaluated. Data were gathered both directly from the patient via personal interview and by reviewing electronic medical records. 

#### 2.1.1. Patient Selection 

The exposure of interest was pneumococcal vaccination status, and the outcome of interest was SARS-CoV-2 pneumonia. Patients whose pneumococcal vaccination status for the recruitment season (ascertained through medical records: health center vaccination record, hospital vaccination record, vaccination card, or medical history) was unknown, were excluded.

#### 2.1.2. SARS-CoV-2 Pneumonia Inclusion Criteria 

Every patient aged between 15 and 64 years who was hospitalized for more than 24 h due to pneumonia and whose chest radiography showed a recent pulmonary infiltrate compatible with pneumonia, alongside one or more of the following signs and symptoms: cough, pleural type chest pain, dyspnea, fever greater than 38 °C, hypothermia less than 35 °C in the last 24 h, or altered respiratory auscultation unexplained by another cause were tested for SARS-CoV-2 infection through a real-time reverse transcription polymerase chain reaction (rRT-PCR).

#### 2.1.3. SARS-CoV-2 Pneumonia Exclusion Criteria 

Patients with nosocomial pneumonia, defined as pneumonia appearing after 48 h or more since admission, were excluded. If the patient was admitted with a diagnosis of pneumonia but had been hospitalized for any cause in the 4 days prior to their current admittance, they were also considered a nosocomial case. Institutionalized patients at the onset of symptoms, patients whose autonomous residence community differed from that of the study participants, and patients with hospital admission of less than 24 h were also excluded.

### 2.2. Data Analysis

A descriptive analysis was undertaken by calculating the frequencies and percentages for the qualitative variables, and the arithmetic mean or median and standard deviation (SD) were used for the quantitative variables. The proportions of categorical variables were compared using the chi-square test for contingency tables or Fisher’s exact test if the number of expected frequencies was over 5. Moreover, a logistic regression was conducted to identify the variables associated with anti-pneumococcal vaccination relative risk, and 95% confidence intervals were calculated in both groups, vaccinated and non-vaccinated. The goodness of fit was verified with the Hosmer–Lemeshow test. All hypothesis tests were two-tailed and, in all the statistical tests, those with a 95% confidence level (*p* < 0.05) were considered significant. The statistical analysis was carried out using IBM SPSS Statistics version 26 (IBM Corp, Armonk, NY, USA).

## 3. Results

The total sample in this study was 300 patients from 15 to 64 years old, primarily from the Andalusia and Catalonia regions. This comprised 70 pneumococcal vaccinated patients and 230 non-vaccinated patients. Of the vaccinated patients, 17 were SARS-CoV-2 pneumonia patients and 53 were CAP by another cause, whereas out of the 230 non-pneumococcal vaccinated patients, 70 were SARS-CoV-2 pneumonia patients and 160 were CAP by another cause. 

### 3.1. Sociodemographic and Health-Related Variables

Sociodemographic and health-related characteristics of patients with/without SARS-CoV-2 pneumonia according to their pneumococcal vaccination status are presented in Table 1. The result of the chi-square test for comparing the difference in population proportions and the results of relative risk analysis and its 95% confidence interval are also presented.

**Figure 1:**
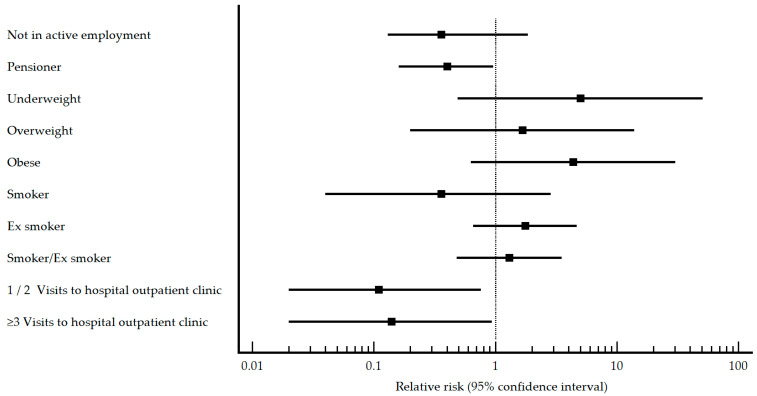
Profile characteristics of relative risk in pneumococcal vaccinated patients with/without SARS-CoV-2.

Among the studied characteristics presented, in the pneumococcal vaccinated group, differences appeared in employment, BMI, smoking habit, and number of visits to hospital outpatient clinics. Pneumonia patients were more likely to work, be obese, be former smokers, and have not visited hospital outpatient clinics. The results of the relative risk analysis for the characteristics that presented differences within the pneumococcal vaccinated group are presented graphically in Figure 1.

When it comes to factors that may be a risk/protective factor for the development of SARS-CoV-2 pneumonia, being a pensioner and visiting hospital outpatient clinics were protective factors, and not using masks in community open spaces was a risk factor. 

In an adjusted analysis, the only factor that appears as protective was visits to hospital outpatient clinics, while visits to primary health care services, being widowed, obese, and not using masks in outdoor open spaces were identified as risk factors.

**Figure 2:**
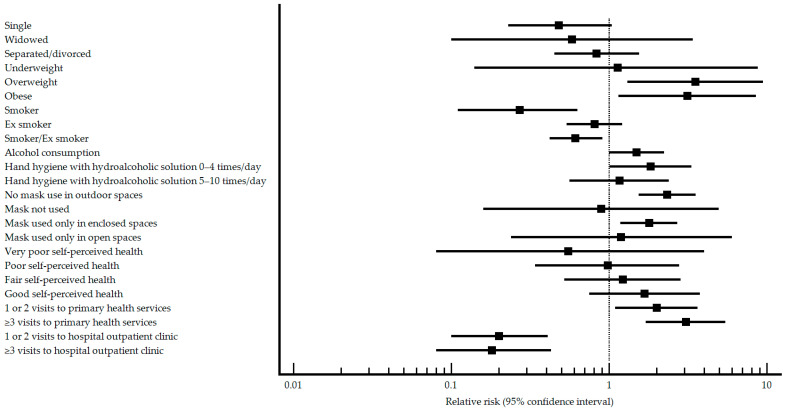
Profile characteristics of relative risk in non-pneumococcal vaccinated patients with/without SARS-CoV-2.

In unvaccinated patients, differences were observed in marital status, BMI, smoking habit, frequency of hand hygiene with hydroalcoholic solution, using masks in outdoor spaces, mask use, self-perceived health, visits to primary health care services, and number of visits to hospital outpatient clinics. Pneumonia patients were more likely to not be married, be overweight or obese, never have smoked, have higher frequency of hand hygiene with hydroalcoholic solution, not using masks in outdoor spaces, only use masks in enclosed spaces, have better perceived health, have visited primary health care services more, and not have visited hospital outpatient clinics. The results of the relative risk analysis for the characteristics that presented differences within the pneumococcal vaccinated group are presented graphically in Figure 2.

When it comes to factors that may be a risk/protective factor for the development of SARS-CoV-2 pneumonia, smoking and visiting hospital outpatient clinics were protective factors, while higher body weight, alcohol consumption, frequent hand hygiene with hydroalcoholic solution, not using mask in outdoor spaces, only using masks in enclosed spaces, and recurrent visits to primary health care services were risk factors.

In the adjusted analysis, the only factor that appears as protective is visits to hospital outpatient clinics, while BMI, alcohol consumption, and not using masks in outdoor open spaces were identified as risk factors.

### 3.2. Comorbidities

Comorbidities of patients with/without SARS-CoV-2 pneumonia, in accordance with their pneumococcal vaccination status, are presented in Table 2. The result of the chi-square test for comparing the difference in population proportions and the results of relative risk analysis and its 95% confidence interval are also presented.

**Figure 3:**
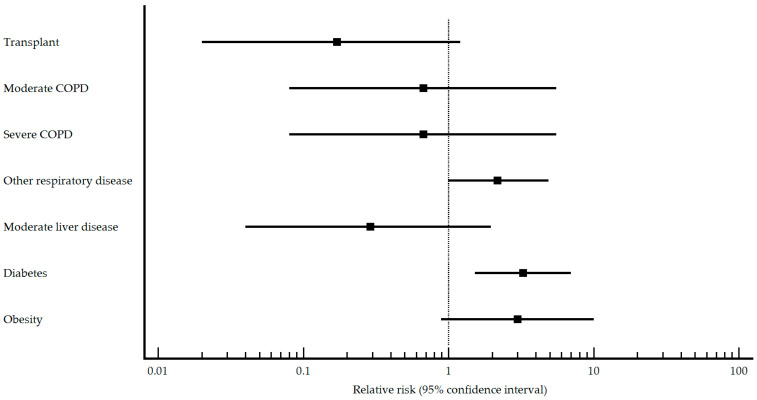
Comorbidities of relative risk in pneumococcal vaccinated patients with/without SARS-CoV-2.

Among the comorbidities examined in the study, in the vaccinated group, differences appear in transplant patients, type of COPD, moderate liver disease, diabetes, and obesity. SARS-CoV-2 pneumonia patients were less likely to have had a transplant, suffer from COPD, have moderate liver disease, have diabetes, or be obese. The results of the relative risk analysis for the comorbidities that presented differences within the pneumococcal vaccinated group are presented graphically in Figure 3.

Concerning comorbidities that may act as protective or risk factors for the development of SARS-CoV-2 pneumonia, in the vaccinated group, none were found, but diabetes or other respiratory disease were identified as risk factors.

**Figure 4:**
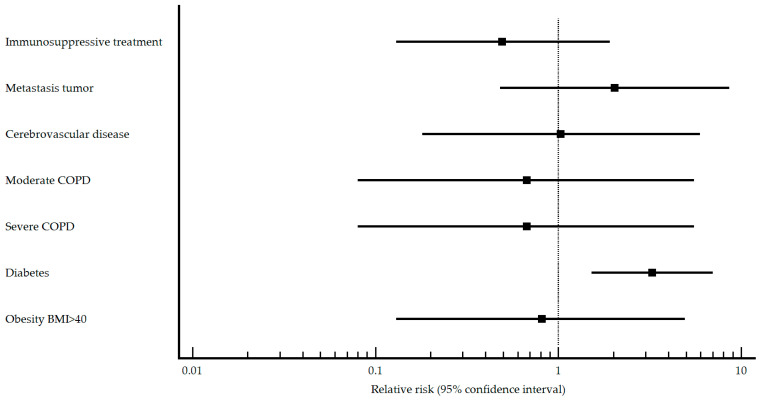
Comorbidities of relative risk in non-pneumococcal vaccinated patients with/without SARS-CoV-2.

For the study involving unvaccinated patients, differences arise in immunosuppressive treatment, metastatic tumors, cerebrovascular disease, type of COPD, diabetes, and obesity with a BMI ≥ 40. SARS-CoV-2 pneumonia patients were less likely to be undergoing immunosuppressive treatment, have metastatic tumors, suffer from severe COPD, have diabetes, or be obese. However, they were more likely to have cerebrovascular disease. The results of the relative risk analysis for the comorbidities that presented differences within the pneumococcal unvaccinated group are presented graphically in Figure 4.

Regarding factors that may act as a risk/protective factor for the development of SARS-CoV-2 pneumonia, undergoing immunosuppressive treatment and having metastatic tumors were protective factors, while cerebrovascular disease, diabetes, and obesity with a BMI ≥ 40 were risk factors.

## 4. Discussion

In this retrospective cohort analysis, using personal characteristics and comorbidities, the difference in the risk of developing SARS-CoV-2 pneumonia between patients 15 and 64 years old with pneumococcal vaccination and patients without pneumococcal vaccination was assessed during the 2020/2021 influenza season, identifying a risk profile for patients in both groups.

The ongoing COVID-19 pandemic highlights that complications and mortality associated with infectious diseases increase with morbidity, as has been seen for SARS-CoV-2 pneumonia. In order to combat this, healthcare providers should promote vaccination against vaccine-preventable diseases, because vaccination would not risk patient safety and health and would improve protection, especially in at-risk groups with a defined risk patient profile. Such strategies might be of significant added value to vaccination.

In this work, the primary analysis evaluated first personal characteristics and then clinical comorbidities, both collectively using the Charlson index and individually, that might increase the risk of developing SARS-CoV-2 pneumonia in patients with and without pneumococcal vaccination.

Previous research has demonstrated that pneumococcal disease is concentrated in a subset of the population with certain medical conditions. For example, among US adults aged 50–64 years, 67% of IPD episodes occurred among those with a chronic medical condition or an immunocompromising condition [27]. This subset comprises 31% of adults aged 50–64 years [28]. In this study, a similar risk profile for developing SARS-CoV-2 pneumonia between both vaccinated and unvaccinated individuals was found. Generally, vaccinated individuals had a lower risk of developing SARS-CoV-2 pneumonia compared to unvaccinated individuals, although they shared similar risk profile characteristics. Therefore, the risk profile is the same, but the level of risk is lower in vaccinated individuals.

In addition to medical conditions, older age, due to immunosenescence and frailty, is a well-recognized risk factor for pneumococcal disease [29]. However, this was not found in this study, probably due to the cutoff age being a relatively low 65 years of age. 

The results showed that the burden of SARS-CoV-2 pneumonia was concentrated in adults with underweight due to deficiency and excess, a history of alcohol consumption, and those who do not frequently use masks in open spaces and who are regular attendees of primary care services. This suggests that they may have an unstable health condition, although their self-assessment of health did not show a higher risk of SARS-CoV-2 pneumonia. Surprisingly, patients who are followed in hospital outpatient clinics, which usually are patients requiring more intense supervision, showed a lower risk of SARS-CoV-2 pneumonia, possibly due to this ongoing surveillance or supervision. 

The presence of comorbidity, especially among non-vaccinated patients, was identified as a significant risk factor for the development of SARS-CoV-2 pneumonia. Among vaccinated patients, a risk of 2.18 (95% CI 1.00–4.88) in patients with a history of respiratory disease was observed. A history of diabetes also increased the risk in both vaccinated and non-vaccinated individuals, with a relative risk of 3.26 (95% CI 1.52–6.99) for vaccinated individuals, while non-vaccinated individuals showed similar results, with a relative risk of 2.58 (95% CI 1.81–3.68).

Identifying the characteristics to be considered among patients for special monitoring is crucial to increase the protection capability of vaccination. This study shows that the patient’s risk profile, personal characteristics, and comorbidities may be useful in patient follow-up after vaccination. Surveillance and the control of patients, as well as ongoing monitoring, can reduce the risk of developing SARS-CoV-2 pneumonia. Additionally, they can help identify modifiable factors, such as body weight and tobacco use, which could be subject to intervention that may help to mitigate the risk of developing SARS-CoV-2 pneumonia. 

This study has several limitations that must be considered. First, the small sample size, and the possibility that disease episodes and risk profiles may have been misclassified or incomplete. However, it is likely that this limitation had a nondifferential impact on disease rates across the vaccinated and non-vaccinated groups, leaving the rate ratios largely unaffected. Second, due to the lack of information on pneumococcal serotypes, it was not possible to assess the proportion of disease caused by serotypes included in pneumococcal vaccines (e.g., PCV13 and PPSV23). Nevertheless, this study allows to evaluate the patient profile associated with SARS-CoV-2 pneumonia development in Spain, which could be considered to improve coverage or increase vaccine effectiveness. The information was collected rigorously, despite the small sample size, which is due to strict criteria and the critical moment of service saturation during the COVID-19 pandemic.

Despite the World Health Organization no longer categorizing SARS-CoV-2 as a public health emergency of international concern, understanding the factors associated with the development of SARS-CoV-2 pneumonia can aid in shaping public health strategies and initiatives. Based on the experience of this study, it would be necessary to consider the personal and clinical characteristics of patients to offer them better protection against SARS-CoV-2 pneumonia.

## 5. Conclusions

This study demonstrates that the risk of developing SARS-CoV-2 pneumonia is high among adults with risk conditions. The findings suggest that vaccination against *S. pneumoniae* could prevent and reduce SARS-CoV-2 pneumonia. Additionally, this study has identified individuals with other medical conditions, such as obesity, underweight, diabetes, and a history of respiratory diseases, who are at an increased risk of developing SARS-CoV-2 pneumonia and could benefit from vaccination and supervision. Future studies quantifying disease risk are needed to evaluate strategies to support vaccinated individuals and reduce the risk of pneumonia development in a way to improve vaccination coverage.

## Figures and Tables

**Table 1:** Sociodemographic and health-related characteristics of patients with/without SARS-CoV-2 pneumonia according to their pneumococcal vaccination status.

	Vaccinated (n = 70, 23.3%)				Not Vaccinated (n = 230, 76.7%)			
	SARS-CoV-2 Pneumonia (n = 17, 24.3%)	No SARS-CoV-2 Pneumonia (n = 53, 75.7%)	*p*-Value	RR (95% CI)	SARS-CoV-2 Pneumonia (n = 70, 30.4%)	No SARS-CoV-2 Pneumonia (n = 160, 69.6%)	*p*-Value	RR (95% CI)
**Age** Median (IQR)	58.00 (9.00)	59.00 (11.00)	0.078	-	53.60 (11.00)	55.00 (45.00)	0.422	-
**Age groups** n (%) <50 years 50–60 years >60 years	4 (23.5) 9 (52.9) 4 (23.5)	12 (22.6) 25 (47.2) 16 (30.2)	0.939 0.685 0.597	Reference 1.06 (0.38–2.93) 0.80 (0.24–2.71)	21 (30.0) 37 (52.9) 12 (17.1)	52 (32.5) 69 (43.1) 39 (24.4)	0.708 0.171 0.221	Reference 1.21 (0.78–1.89) 0.82 (0.44–1.51)
**Gender** n (%) Female Male	3 (17.6) 14 (82.4)	18 (34.0) 35 (66.0)	0.203 0.203	Reference 2.00 (0.64–6.24)	28 (40.0) 42 (60.0)	65 (40.6) 95 (59.4)	0.932 0.932	Reference 1.02 (0.68–1.52)
**Level of education** n (%) University Secondary or PT Primary	2 (11.8) 5 (29.4) 10 (58.8)	7 (13.2) 9 (17.0) 37 (69.8)	0.882 0.270 0.404	Reference 1.61 (0.39–6.58) 0.96 (0.25–3.66)	13 (18.6) 11 (15.7) 46 (65.7)	29 (18.1) 41 (25.6) 90 (56.3)	0.928 0.099 0.183	Reference 0.68 (0.34–1.37) 1.09 (0.66–1.49)
**Employment status** n (%) Not in active employment Pensioner Worker	2 (11.8) 6 (35.3) 9 (52.9)	8 (15.4) 31 (59.6) 13 (25.0)	0.160 0.083 **0.033**	0.36 (0.13–1.86) **0.40 (0.16–0.96)** Reference	9 (12.9) 12 (17.1) 49 (70.0)	37 (23.6) 29 (18.5) 91 (58.0)	0.064 0.800 0.086	0.56 (0.30–1.05) 0.84 (0.49–1.42) Reference
**Works in an educational center** n (%) No Yes	11 (73.3) 4 (26.7)	32 (64.0) 18 (36.0)	0.484 0.484	Reference 0.71 (0.26–1.98)	26 (37.7) 43 (62.3)	52 (33.8) 102 (66.2)	0.570 0.570	Reference 0.89 (0.60–1.33)
**Marital status** n (%) Single Married Widowed Separated/divorced	1 (5.9) 11 (64.7) 1 (5.9) 4 (23.5)	8 (15.1) 34 (64.2) 1 (1.9) 10 (18.9)	0.328 0.970 0.394 0.682	0.45 (0.07–3.09) Reference 2.05 (0.47–8.97) 1.17 (0.44–3.10)	6 (8.6) 55 (78.6) 1 (1.4) 8 (11.4)	30 (18.9) 105 (66.0) 4 (2.5) 20 (12.6)	**0.049** 0.056 0.599 0.799	0.48 (0.23–1.04) Reference 0.58 (0.10–3.40) 0.83 (0.45–1.55)
**Coexistence** n (%) Living alone With cohabitants	4 (23.5) 13 (76.5)	12 (22.6) 41 (77.4)	0.939 0.939	1.04 (0.39–2.74) Reference	4 (5.7) 66 (94.3)	18 (11.3) 142 (88.8)	0.186 0.186	0.57 (0.23–1.42) Reference
**Body Mass Index** n (%) Underweight Normal weight Overweight Obese	0 (0) 1 (9.1) 3 (27.3) 7 (63.6)	1 (2.9) 9 (26.5) 15 (44.1) 9 (26.5)	0.481 0.136 0.222 **0.006**	5.00 (0.49–50.83) Reference 1.67 (0.20–13.98) 4.38 (0.63–30.46)	0 (0) 4 (10.5) 18 (47.4) 16 (42.1)	7 (7.2) 32 (33.0) 28 (28.9) 30 (30.9)	**0.022** **<0.001** **0.007** 0.100	1.13 (0.14–8.77) Reference **3.52 (1.31–9.49)** **3.13 (1.15–8.55)**
**Smoking habit** n (%) Never smoked Smoker Ex-smoker Smoker/ex-smoker	4 (23.5) 0 (0) 13 (76.5) 13 (76.5)	16 (30.2) 13 (24.5) 24 (45.3) 37 (69.8)	0.597 **0.025** **0.026** 0.825	Reference 0.36 (0.04–2.87) 1.76 (0.66–4.68) 1.30 (0.48–3.51)	38 (54.3) 5 (7.1) 27 (38.6) 32 (45.7)	59 (36.9) 43 (26.9) 58 (36.3) 101 (63.1)	**0.014** **<0.001** 0.740 **0.013**	Reference **0.27 (0.11–0.63)** 0.81 (0.54–1.21) **0.61 (0.42–0.91)**
**Alcohol consumption** n (%) No Yes	10 (58.8) 7 (41.2)	41 (77.4) 12 (22.6)	0.136 0.136	Reference 1.88 (0.84–4.22)	48 (68.6) 22 (31.4)	128 (80.0) 32 (20.0)	0.061 0.061	Reference **1.49 (1.00–2.23)**
**Frequency of hand washing** n (%) 0–4 times/day 5–10 times/day >10 times/day	5 (29.4) 7 (41.2) 5 (29.4)	14 (26.4) 21 (39.6) 18 (34.0)	0.810 0.907 0.727	1.21 (0.21–3.57) 1.15 (0.42–3.15) Reference	20 (28.6) 32 (45.7) 18 (25.7)	52 (32.5) 64 (40.0) 44 (27.5)	0.558 0.421 0.778	0.96 (0.56–1-64) 1.15 (0.71–1.86) Reference
**Frequency of hand hygiene with hydroalcoholic solution** n (%) 0–4 times/day 5–10 times/day >10 times/day	9 (52.9) 3 (17.6) 5 (29.4)	28 (52.8) 16 (30.2) 9 (17.0)	0.671 0.313 0.270	0.68 (0.28–1.68) 0.44 (0.13–1.55) Reference	47 (67.1) 13 (18.6) 10 (14.3)	79 (49.4) 42 (26.3) 39 (24.4)	**0.013** 0.209 0.086	**1.83 (1.01–3.32)** 1.16 (0.56–2.40) Reference
**Mask use in enclosed spaces** n (%) No Yes	0 (0) 17 (100.0)	1 (1.9) 52 (98.1)	0.570 0.570	Reference 0.49 (0.12–2.09)	1 (1.4) 69 (98.6)	5 (3.1) 155 (96.9)	0.456 0.456	Reference 1.85 (0.31–11.18)
**Mask use in outdoor spaces** n (%) No Yes	6 (37.5) 10 (62.5)	14 (28.0) 36 (72.0)	0.462 0.462	1.38 (0.58–3.28) Reference	38 (59.4) 26 (40.6)	46 (29.9) 108 (70.1)	**<0.001** **<0.001**	**2.33 (1.54–3.54)** Reference
**Mask use in community open spaces** n (%) No Yes	4 (23.5) 13 (76.5)	4 (7.8) 47 (92.2)	0.081 0.081	2.31 (1.00–5.36) Reference	15 (21.7) 54 (78.3)	21 (13.4) 136 (86.6)	0.114 0.114	1.47 (0.94–2.29) Reference
**Mask use** n (%) Not used Only in enclosed spaces Only in open spaces In open and enclosed spaces	0 (0) 3 (17.6) 0 (0) 14 (82.4)	0 (0) 5 (9.4) 1 (1.9) 47 (88.7)	- 0.358 0.570 0.502	2.18 (0.51–9.38) 1.63 (0.60–4.47) 2.18 (0.51–9.38) Reference	0 (0) 16 (22.9) 0 (0) 54 (77.1)	3 (1.9) 16 (10.0) 2 (1.3) 139 (86.9)	0.247 **0.010** 0.339 0.063	0.89 (0.16–4.95) **1.79 (1.18–2.70)** 1.19 (0.24–6.00) Reference
**Mask type** n (%) Hygienic, reusable Hygienic, non-reusable Surgical Self-filtering FFP2	0 (0) 0 (0) 14 (82.4) 3 (17.6)	3 (5.7) 1 (1.9) 32 (60.4) 17 (32.1)	0.318 0.570 0.100 0.253	1.67 (0.23–12.22) 3.33 (0.59–18.89) 2.03 (0.65–6.29) Reference	2 (2.9) 4 (5.7) 46 (65.7) 18 (25.7)	12 (7.6) 4 (2.5) 90 (57.3) 51 (32.5)	0.174 0.224 0.233 0.303	0.55 (0.14–2.10) 1.92 (0.86–4.26) 1.30 (0.82–2.06) Reference
**Frequency of mask change** n (%) >2 times/day 1 time/day 3 times/week 2 times/week 1 time/week	0 (0) 10 (58.8) 5 (29.4) 2 (11.8) 0 (0)	6 (11.3) 22 (41.5) 14 (26.4) 6 (11.3) 5 (9.4)	0.150 0.216 0.810 0.955 0.193	Reference 2.19 (0.33–14.42) 1.84 (0.26–13.14) 1.75 (0.20–15.41) 1.17 (0.09–14.92)	7 (10.0) 31 (44.3) 12 (17.1) 10 (14.3) 10 (14.3)	18 (11.5) 62 (39.5) 27 (17.2) 27 (17.2) 23 (14.6)	0.739 0.497 0.986 0.585 0.953	Reference 1.19 (0.60–2.38) 1.10 (0.50–2.41) 0.97 (0.42–2.20) 1.08 (0.48–2.44)
**Self-perceived health** n (%) Very poor Poor Fair Good Very good	1 (5.9) 2 (11.8) 8 (47.1) 4 (23.5) 2 (11.8)	3 (5.7) 7 (13.2) 19 (35.8) 22 (41.5) 2 (3.8)	0.976 0.882 0.408 0.185 0.221	0.50 (0.07–2.55) 0.44 (0.09–2.13) 0.59 (0.19–1.85) 0.31 (0.08–1.17) Reference	1 (1.4) 6 (8.6) 21 (30.0) 37 (52.9) 5 (7.1)	7 (4.4) 21 (13.1) 55 (34.4) 60 (37.5) 17 (10.6)	0.255 0.330 0.515 **0.030** 0.407	0.55 (0.08–4.02) 0.98 (0.34–2.78) 1.22 (0.52–2.85) 1.68 (0.75–3.78) Reference
**Visits to primary health services** n (%) 0 1–2 ≥3	5 (29.4) 6 (35.3) 6 (35.3)	25 (47.2) 16 (30.2) 12 (22.6)	0.200 0.696 0.301	Reference 1.64 (0.57–4.68) 2.00 (0.71–5.62)	12 (17.1) 28 (40.0) 30 (42.9)	65 (40.6) 62 (38.8) 33 (20.6)	**<0.001** 0.864 **<0.001**	Reference **2.00 (1.09–3.65)** **3.06 (1.71–5.46)**
**Visits to specialist** n (%) 0 1–2 ≥3	9 (52.9) 4 (23.5) 4 (23.5)	29 (54.7) 13 (24.5) 11 (20.8)	0.898 0.934 0.815	Reference 0.99 (0.35–2.78) 1.17 (0.30–4.60)	43 (61.4) 21 (30.0) 6 (8.6)	102 (63.7) 33 (20.6) 25 (15.6)	0.740 0.122 0.153	Reference 1.31 (0.86–1.99) 0.57 (0.22–1.49)
**Visits to hospital outpatient clinic** n (%) 0 1–2 ≥3	16 (94.1) 0 (0) 1 (5.9)	19 (35.8) 19 (35.8) 15 (28.3)	**<0.001** **0.004** 0.057	Reference **0.11 (0.02–0.76)** **0.14 (0.02–0.94)**	58 (82.9) 7 (10.0) 5 (7.1)	53 (33.1) 60 (37.5) 47 (29.4)	**<0.001** **<0.001** **<0.001**	Reference **0.20 (0.10–0.41)** **0.18 (0.08–0.43)**
**Admission to hospital** n (%) 0–2 ≥3	17 (100.0) 0 (0)	51 (96.2) 2 (3.8)	0.418 0.418	Reference 1.33 (0.26–6.96)	69 (98.6) 1 (1.4)	154 (96.3) 6 (3.8)	0.348 0.348	Reference 0.46 (0.07–2.86)

95% CI: 95% confidence interval; IQR: interquartile range; RR: relative risk; SARS-CoV-2: severe acute respiratory syndrome coronavirus 2.

**Table 2:** Comorbidities of patients with/without SARS-CoV-2 pneumonia according to their pneumococcal vaccination status.

	Vaccinated (n = 70, 23.3%)				Not Vaccinated (n = 230, 76.7%)			
	SARS-CoV-2 Pneumonia (n = 17, 24.3%)	No SARS-CoV-2 Pneumonia (n = 53, 75.7%)	*p*-Value	RR (95% CI)	SARS-CoV-2 Pneumonia (n = 70, 30.4%)	No SARS-CoV-2 Pneumonia (n = 160, 69.6%)	*p*-Value	RR (95% CI)
**COMORBIDITY** n (%) No Yes	0 (0) 17 (100.0)	1 (1.9) 52 (98.1)	0.570	Reference 0.49 (0.12–2.09)	1 (1.4) 69 (98.6)	11 (6.9) 149 (93.1)	0.085	Reference 3.80 (0.58–25.06)
**Charlson comorbidity index** n (%) None Low High	6 (35.3) 6 (35.3) 5 (29.4)	14 (26.4) 8 (15.1) 31 (58.5)	0.078	Reference 1.43 (0.58–3.52) 0.46 (0.16–1.33)	19 (27.1) 34 (48.6) 17 (24.3)	49 (30.6) 27 (16.9) 84 (52.5)	<0.001	Reference 1.99 (1.28–3.10) 0.60 (0.34–1.07)
**Chronic respiratory failure** n (%) No Yes	14 (82.4) 3 (17.6)	47 (88.7) 6 (11.3)	0.502	Reference 1.45 (0.52–4.08)	68 (97.1) 2 (2.9)	153 (95.6) 7 (4.4)	0.592	Reference 0.72 (0.21–2.49)
**Pneumonia in the last 2 years** n (%) No Yes	15 (93.8) 1 (6.3)	50 (94.3) 3 (5.7)	0.939	Reference 1.08 (0.19–6.26)	69 (98.6) 1 (1.4)	154 (96.3) 6 (3.8)	0.348	Reference 0.46 (0.07–2.86)
**Cellular/humoral immunity** n (%) No Yes	17 (100.0) 0 (0)	50 (94.3) 3 (5.7)	0.318	Reference 0.99 (0.17–5.65)	70 (100.0) 0 (0)	155 (96.9) 5 (3.1)	0.137	Reference 0.54 (0.09–3.24)
**Immunosuppressive treatment** n (%) No Yes	15 (88.2) 2 (11.8)	40 (75.5) 13 (24.5)	0.270	Reference 0.49 (0.13–1.91)	69 (98.6) 1 (1.4)	138 (86.8) 21 (13.2)	**0.005**	Reference **0.14 (0.02–0.93)**
**Transplant** n (%) No Yes	17 (100.0) 0 (0)	36 (67.9) 17 (32.1)	**0.008**	Reference 0.17 (0.02–1.21)	69 (98.6) 1 (1.4)	155 (96.9) 5 (3.1)	0.456	Reference 0.54 (0.09–3.27)
**HIV** n (%) No Yes	16 (94.1) 1 (5.9)	52 (98.1) 1 (1.9)	0.364	Reference 2.13 (0.50–9.07)	70 (100.0) 0 (0)	158 (98.7) 2 (1.3)	0.339	Reference 1.09 (0.22–5.44)
**AIDS** n (%) No Yes	17 (100.0) 0 (0)	53 (100.0) 0 (0)	1	Reference 2.06 (0.48–8.74)	70 (100.0) 0 (0)	158 (98.7) 2 (1.3)	0.339	Reference 1.09 (0.22–5.44)
**Metastasic tumor** n (%) No Yes	17 (100.0) 0 (0)	52 (98.1) 1 (1.9)	0.570	Reference 2.03 (0.48–8.62)	69 (98.6) 1 (1.4)	138 (86.3) 22 (13.8)	**0.004**	Reference **0.13 (0.02–0.90)**
**Non metastatic tumor** n (%) No Yes	16 (94.1) 1 (5.9)	47 (88.7) 6 (11.3)	0.521	Reference 0.56 (0.09–3.63)	64 (91.4) 6 (8.6)	136 (85.0) 24 (15.0)	0.186	Reference 0.63 (0.30–1.31)
**Lymphoma** n (%) No Yes	17 (100.0) 0 (0)	50 (94.3) 3 (5.7)	0.318	Reference 0.99 (0.17–5.65)	69 (98.6) 1 (1.4)	155 (96.9) 5 (3.1)	0.456	Reference 0.54 (0.09–3.27)
**Leukemia** n (%) No Yes	17 (100.0) 0 (0)	53 (100.0) 0 (0)	1	Reference 2.06 (0.48–8.74)	69 (98.6) 1 (1.4)	154 (96.3) 6 (3.8)	0.348	Reference 0.46 (0.07–2.86)
**Chronic renal failure** n (%) No Yes	16 (94.1) 1 (5.9)	45 (84.9) 8 (15.1)	0.328	Reference 0.42 (0.06–2.82)	70 (100.0) 0 (0)	155 (96.9) 5 (3.1)	0.137	Reference 0.54 (0.09–3.24)
**Asplenia** n (%) No Yes	17 (100.0) 0 (0)	51 (96.2) 2 (3.8)	0.418	Reference 1.33 (0.26–6.96)	69 (98.6) 1 (1.4)	160 (100.0) 0 (0)	0.135	Reference 1.66 (0.41–6.73)
**Myocardial infarction** n (%) No Yes	16 (94.1) 1 (5.9)	50 (94.3) 3 (5.7)	0.976	Reference 1.03 (0.18–5.94)	69 (98.6) 1 (1.4)	150 (93.7) 10 (6.3)	0.111	Reference 0.29 (0.04–1.89)
**Congestive heart failure** n (%) No Yes	16 (94.1) 1 (5.9)	45 (84.9) 8 (15.1)	0.328	Reference 0.42 (0.06–2.82)	68 (97.1) 2 (2.9)	147 (91.9) 13 (8.1)	0.142	Reference 0.42 (0.11–1.55)
**Peripheral vascular disease** n (%) No Yes	16 (94.1) 1 (5.9)	47 (88.7) 6 (11.3)	0.521	Reference 0.56 (0.09–3.63)	66 (94.3) 4 (5.7)	151 (94.4) 9 (5.6)	0.976	Reference 1.01 (0.44–2.34)
**Other cardiac diseases** n (%) No Yes	12 (70.6) 5 (29.4)	37 (69.8) 16 (30.2)	0.950	Reference 0.97 (0.39–2.41)	42 (60.0) 28 (40.0)	113 (70.6) 47 (29.4)	0.115	Reference 1.38 (0.93–2.04)
**Cerebrovascular disease** n (%) No Yes	16 (94.1) 1 (5.9)	50 (94.3) 3 (5.7)	0.976	Reference 1.03 (0.18–5.94)	65 (92.9) 5 (7.1)	159 (99.4) 1 (0.6)	**0.004**	Reference **2.87 (1.90–4.34)**
**COPD** n (%) No Yes	16 (94.1) 1 (5.9)	48 (90.6) 5 (9.4)	0.656	Reference 0.67 (0.11–4.19)	66 (94.3) 4 (5.7)	153 (95.6) 7 (4.4)	0.672	Reference 1.21 (0.54–2.71)
**COPD type** n (%) Mild Moderate Severe	0 (0) 1 (100.0) 0 (0)	0 (0) 2 (50.0) 2 (50.0)	- **<0.001** **<0.001**	Reference 0.67 (0.08–5.54) 0.67 (0.08–5.54)	1 (33.3) 2 (66.7) 0 (0)	1 (20.0) 3 (60.0) 1 (20.0)	**0.030** 0.336 **<0.001**	Reference 0.80 (0.14–4.62) 1.00 (0.14–7.10)
**Other respiratory disease** n (%) No Yes	11 (64.7) 6 (35.3)	45 (84.9) 8 (15.1)	0.072	Reference **2.18 (1.00–4.88)**	62 (88.6) 8 (11.4)	148 (92.5) 12 (7.5)	0.335	Reference 1.35 (0.76–2.41)
**Neurological disease** n (%) No Yes	17 (100.0) 0 (0)	51 (96.2) 2 (3.8)	0.418	Reference 1.33 (0.26–6.96)	68 (97.1) 2 (2.9)	150 (93.7) 10 (6.3)	0.289	Reference 0.53 (0.15–1.92)
**Dementia** n (%) No Yes	17 (100.0) 0 (0)	53 (100.0) 0 (0)	1	Reference 2.06 (0.48–8.74)	70 (100.0) 0 (0)	158 (98.7) 2 (1.3)	0.339	Reference 1.09 (0.22–5.44)
**Neuromuscular disease** n (%) No Yes	17 (100.0) 0 (0)	51 (96.2) 2 (3.8)	0.418	Reference 1.33 (0.26–6.96)	67 (95.7) 3 (4.3)	158 (98.7) 2 (1.3)	0.156	Reference 2.01 (0.96–4.24)
**Chronic kidney disease** n (%) No Yes	16 (94.1) 1 (5.9)	46 (86.8) 7 (13.2)	0.414	Reference 0.48 (0.07–3.18)	68 (97.1) 2 (2.9)	149 (93.1) 11 (6.9)	0.229	Reference 0.49 (0.14–1.78)
**Moderate liver disease** n (%) No Yes	17 (100.0) 0 (0)	42 (79.2) 11 (20.8)	**0.042**	Reference 0.29 (0.04–1.97)	69 (98.6) 1 (1.4)	148 (92.5) 12 (7.5)	0.066	Reference 0.24 (0.04–1.61)
**Mild liver disease** n (%) No Yes	17 (100.0) 0 (0)	50 (94.3) 3 (5.7)	0.318	Reference 0.99 (0.17–5.75)	66 (94.3) 4 (5.7)	151 (94.4) 9 (5.6)	0.976	Reference 1.01 (0.44–2.34)
**Diabetes** n (%) No Yes	9 (52.9) 8 (47.1)	46 (86.8) 7 (13.2)	**0.003**	Reference **3.26 (1.52–6.99)**	43 (61.4) 27 (38.6)	142 (88.7) 18 (11.3)	**<0.001**	Reference **2.58 (1.81–3.68)**
**Connective tissue disease** n (%) No Yes	17 (100.0) 0 (0)	51 (96.2) 2 (3.8)	0.418	Reference 1.33 (0.26–6.96)	70 (100.0) 0 (0)	155 (96.9) 5 (3.1)	0.137	Reference 0.54 (0.09–3.24)
**Peptic ulcer** n (%) No Yes	16 (94.1) 1 (5.9)	52 (98.1) 1 (1.9)	0.394	Reference 2.13 (0.50–9.07)	66 (94.3) 4 (5.7)	153 (95.6) 7 (4.4)	0.672	Reference 1.21 (0.54–2.71)
**Hemophilia** n (%) No Yes	17 (100.0) 0 (0)	53 (100.0) 0 (0)	1	Reference 2.06 (0.48–8.74)	69 (98.6) 1 (1.4)	160 (100.0) 0 (0)	0.135	Reference 1.66 (0.41–6.73)
**Anemia** n (%) No Yes	17 (100.0) 0 (0)	50 (94.3) 3 (5.7)	0.318	Reference 0.99 (0.17–5.65)	67 (95.7) 3 (4.3)	155 (96.9) 5 (3.1)	0.648	Reference 1.24 (0.50–3.11)
**Obesity** n (%) No Yes	3 (27.3) 8 (72.5)	25 (59.5) 17 (40.5)	**0.022**	Reference 2.99 (0.89–10.04)	48 (68.6) 22 (31.4)	127 (79.4) 33 (20.6)	0.078	Reference 1.46 (0.97–2.18)
**Obesity BMI ≥ 40** n (%) No Yes	16 (94.1) 1 (5.9)	49 (92.5) 4 (7.5)	0.825	Reference 0.81 (0.13–4.93)	62 (88.6) 8 (11.4)	153 (95.6) 7 (4.4)	**0.049**	Reference **1.85 (1.10–3.10)**

AIDS: acquired immunodeficiency syndrome; COPD: chronic obstructive pulmonary disease; HIV: human immunodeficiency virus.

## Data Availability

The data are not publicly available due to privacy and ethical restrictions.
